# Benchmarking a Local Schema-Constrained Large Language Model Pipeline for Abstract Screening and Evidence Mapping

**DOI:** 10.7759/cureus.111193

**Published:** 2026-06-20

**Authors:** Alessandro Serretti

**Affiliations:** 1 Psychiatry, Università degli Studi di Enna Kore, Enna, ITA; 2 Psychiatry, Oasi Research Institute-IRCCS, Troina, ITA

**Keywords:** brain imaging, large language models, mapping evidence, psychiatry, scoping review

## Abstract

Background

The growth of biomedical literature increasingly exceeds the capacity of manual evidence synthesis. Large language models (LLMs) may support abstract screening and structured extraction, but many current workflows depend on proprietary cloud APIs, creating challenges for governance, reproducibility, and scalable deployment.

Methods

I developed a fully local, open-weight, schema-constrained pipeline (gpt-oss-20b, deployed via Ollama on Apple M1 Max) for title/abstract-based scoping workflows. The pipeline combined deterministic metadata filtering, LLM-assisted screening, and structured abstract extraction. Performance was benchmarked against three published systematic reviews (ketamine/neuroimaging; clozapine/suicidality; clozapine patient/caregiver perspectives) using precision, recall, and F1 against reference inclusion sets. I also report audit-adjusted estimates (i.e., performance metrics recalculated after manual full-text adjudication of discrepant records) alongside standard reference-set performance.

Results

In the ketamine/neuroimaging benchmark, the pipeline retained all 41 studies included in the original review; after audit adjustment, recall was 100.0% (46/46), accuracy 99.4% (156/157), precision 97.9% (46/47), and F1 98.9%. For clozapine/suicidality, recall was 79.3% (46/58), and F1 was 76.0%, with missed studies largely attributable to missing or non-informative abstracts. For clozapine patient/caregiver perspectives, recall was 88.9% (56/63), and F1 was 83.6%, with similar abstract-level constraints. Abstract-level extraction recovered audited metadata fields without detected errors and generated evidence maps that were thematically concordant with the main narrative structure of the reference reviews.

Conclusions

As a proof-of-concept, a fully local LLM pipeline can support scalable and auditable abstract-based scoping and high-level evidence mapping. Because performance was benchmarked against three reviews with partly audit-adjusted reference sets, the findings require confirmation in larger, independently adjudicated evaluations. Random human audit remains advisable, and expert full-text synthesis remains necessary when abstracts are non-informative or when mechanistic precision is required.

## Introduction

The biomedical literature is expanding faster than traditional evidence-synthesis workflows can reasonably absorb. Systematic reviews (SRs) remain the methodological gold standard for informing clinical decision-making, yet their standard workflow scales poorly: comprehensive screening, full-text eligibility assessment, and structured extraction routinely require months of manual labor. This time burden encourages narrowly focused questions and discourages broader, clinically informative syntheses. The result is a structural bottleneck in which large fractions of the literature remain effectively unsynthesized, and “whole-field” evidence maps that could guide hypothesis generation, trial design, and translational prioritization are rarely feasible under purely manual constraints.

Large Language Models (LLMs) offer a plausible route around this bottleneck because they can perform high-throughput natural-language classification and schema-constrained extraction directly from papers. Recent studies indicate that generative models can achieve promising performance in the screening phase [1,2], with reported mean precision of 83.0% and recall of 86.0% [3]. However, most published implementations have focused on retrieval and/or screening rather than end-to-end synthesis [4,5]. LLM-based approaches have also been explored across a range of literature review contexts - including cross-study summarization, screening calibration, benchmark dataset development, and broader clinical commentary - using both cloud-based and local architectures [6-11]. Together, these studies support the feasibility of LLM-assisted reviewing, while also highlighting the gap between proof-of-concept performance and deployable scientific workflows.

One reason for this gap is the reliance of many operational pipelines on proprietary cloud platforms. This dependence introduces several recurring problems: governance and privacy concerns when clinical or proprietary text is transmitted to external services; escalating costs and throughput limits when scaling to thousands of records; and compromised reproducibility, as proprietary model versions, decoding strategies, and safety filters are subject to opaque updates. API latency and rate limits further impose a hard ceiling on analysis volume, undermining the very scalability that motivates automation.

A local, open-weight alternative is therefore increasingly attractive: LLMs can be run on-device within a reproducible computational environment, using fixed model artifacts, explicit prompts, and an auditable decision trail. This architecture supports data sovereignty, reduces marginal inference costs, and enables deterministic logging of screening decisions and extraction outputs. It also enables high-throughput scoping: rather than narrowing the question to match available human labor, the computational workflow can expand to cover broader evidence fields while preserving traceability and reproducibility. Recent reports suggest good performance in both screening and extraction using relatively small 3B models [1], and ongoing model improvements may further improve reliability [12]. An additional potential advantage is multilingual capability, which can extend coverage beyond English-centric studies [13].

A central design choice in scalable automation is the level of text to be analyzed. Full-text processing is necessary for mechanistic precision (e.g., coordinate-level neuroimaging findings, detailed analytic pipelines, adverse event tables), but it remains constrained by access barriers, heterogeneous formatting, and the technical fragility of PDF parsing. Abstract-level processing, in contrast, is widely available at scale and may be sufficient to recover the dominant narrative structure of a field: which constructs are studied, which regions or networks recur in imaging studies, and what qualitative directionality is repeatedly reported. As a trade-off, abstracts may compress conditionality (e.g., baseline predictors versus longitudinal change), collapse anatomical granularity (e.g., broad labels), and omit many methodological details. Consequently, abstract-based automation is well-suited to scoping and hypothesis generation, but it cannot replace full-text expert synthesis when the objective is coordinate-level inference or effect-size-level mechanistic integration.

Accordingly, I aimed to test a fully local, open-weight, schema-constrained LLM pipeline for abstract-based systematic scoping reviews by benchmarking retrieval, structured extraction, and high-level evidence mapping against three published expert systematic reviews, while characterizing the trade-off between scalability and information loss inherent to abstract-only synthesis. Here I use ‘scoping’ to denote high-throughput mapping of constructs and qualitative patterns from titles/abstracts, rather than full-text risk-of-bias assessment or effect-size meta-analysis. I benchmark against published systematic reviews as reference standards for inclusion sets and narrative structure, while explicitly recognizing that abstract-only pipelines cannot replicate full-text eligibility and mechanistic synthesis.

## Materials and methods

Local computational setting

The pipeline was executed fully locally, without external APIs, on an Apple M1 Max workstation (64 GB unified memory) using Ollama (v 0.21.0) for on-device inference. I selected gpt-oss-20b among available models as the only open-weight model that could be executed locally in a stable and natively supported configuration within the available hardware constraints, without requiring additional runtime quantization or format conversion. This ensured consistent inference behavior and reproducible structured outputs across runs. I avoided QLoRA-based instruction tuning and instead used a frozen, pre-trained LLM in a zero-shot, prompt-driven setting to ensure methodological reproducibility and to minimize the risk of task-induced bias introduced by fine-tuning. Unless otherwise specified, inference used near-deterministic decoding (temperature 0.01) with a context window of 4096 tokens, top_p 0.1, top_k 10, repeat_penalty 1.1. System prompts were specified for each LLM task. The computational stack was implemented in Python 3.12 and executed from VS Code. Reproducibility was supported through a modular script architecture, explicit external configuration files (Extraction JSON), and append-only record keeping (JSONL logs) to preserve full provenance of decisions, intermediate outputs, and downstream transformations.

**Figure 1:**
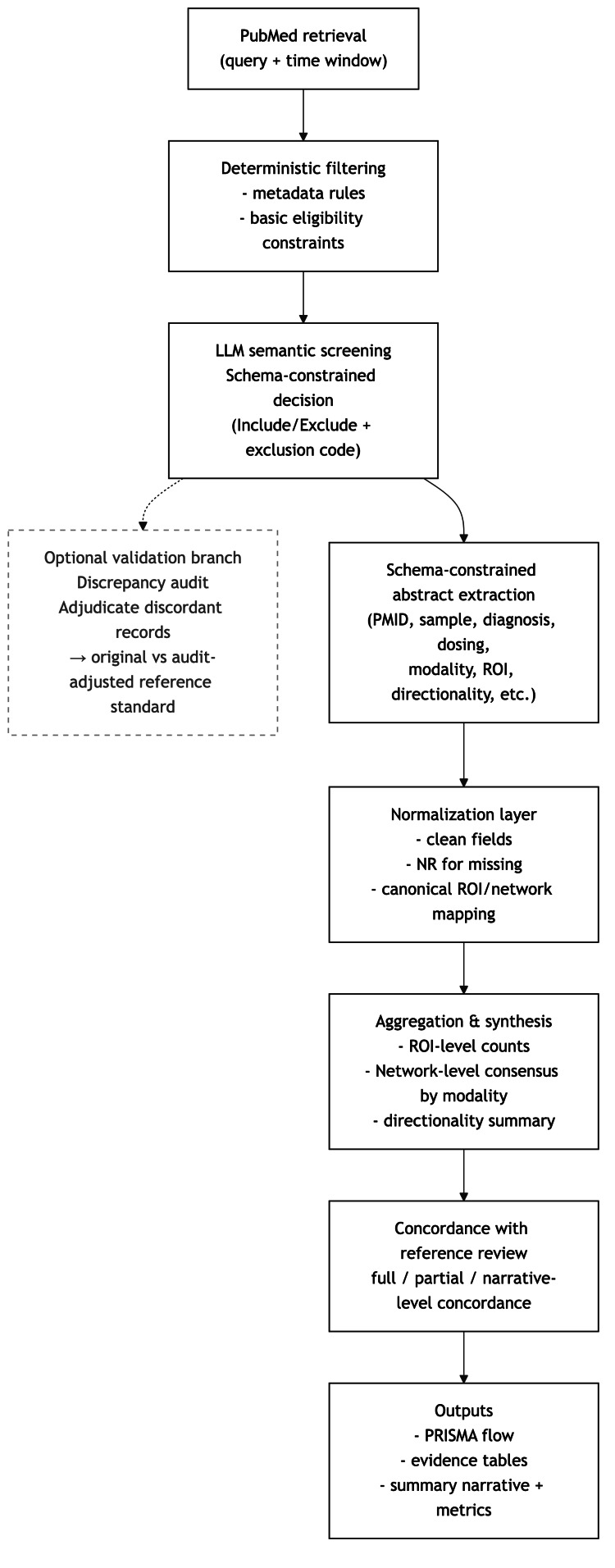
Overview of the LLM-driven abstract-level evidence synthesis pipeline The figure is a conceptual workflow schematic (it does not depict study data); it was authored as a flowchart in the Mermaid diagramming language and rendered to a high-resolution image with the Mermaid command-line interface (mermaid-cli/mmdc, v11.4.2). The diagram definition is provided with the analysis code in the public repository.

The pipeline was designed as a modular sequence of Python scripts and LLM actions that transform raw bibliographic records into structured, analysis-ready outputs (Figure 1). Each stage writes standardized intermediate artifacts to disk, enabling checkpointing, re-runs from arbitrary stages, and transparent auditing (https://github.com/svonte/Scoping-Systematic).

The workflow comprised three stages: (i) programmatic retrieval from PubMed using the same queries and date windows as each reference review; (ii) deterministic metadata filtering to remove clearly ineligible records (e.g., non-human studies; editorials/letters/comments/errata); and (iii) LLM-based title/abstract screening with schema-constrained outputs (Include/Exclude plus standardized exclusion codes). For this benchmark evaluation, retrieval was restricted to PubMed to match the reference searches and minimize retrieval-source bias; duplicate removal was therefore not required. Semantic screening of titles/abstracts was performed using a zero-shot classification strategy with explicit schema enforcement. While few-shot prompting is often preferred for complex reasoning, I selected zero-shot to prevent the introduction of selection bias via exemplar choice [14]. To minimize prompt-induced selection bias, the model was provided only with (i) the eligibility criteria (externalized in a domain-specific criteria file) and (ii) the record text (title and abstract, plus minimal bibliographic metadata when required for disambiguation). Outputs were constrained to a strict JSON object containing: (a) decision (Include/Exclude) and (b) standardized exclusion reason_code aligned with PRISMA-style reporting. All decisions were captured in an append-only, timestamped audit trail, ensuring that screening history remains reconstructable and resistant to silent overwriting. For neuroimaging studies, extracted ROI mentions were mapped to a predefined network taxonomy (DMN/FPN/SN/Limbic/SCN) via an allowlist-based mapping layer; ambiguous items were retained as unmapped. Finally, high-level synthesis aggregated directionality and thematic recurrence using predefined rules tailored to each benchmark.

Benchmarking and metrics

I benchmarked performance against three published systematic reviews representing distinct abstract informativeness profiles: ketamine-neuroimaging [15], clozapine-suicidality [16], and clozapine patient/caregiver perspectives [17]. The three reviews were selected because they addressed complementary topics within psychiatry, used PubMed-based search strategies that could be replicated programmatically, were published in Q1 journals, and fell within the author’s area of methodological and clinical expertise. Given the intrinsic limitations of abstract-only inputs, the evaluation emphasized the pipeline’s intended use case: whether a schema-constrained, abstract-derived evidence map can reproduce the principal conclusions and dominant qualitative signals of expert syntheses, rather than attempting one-to-one replication of granular full-text details. Accordingly, the evaluation combined (i) study-level overlap with the reference inclusion sets and (ii) thematic/directional concordance between the pipeline-derived summaries and the reference reviews’ main narrative claims. Discrepant records (pipeline-retained studies absent from the reference review and reference-included studies not retained by the pipeline) were manually adjudicated against the prespecified eligibility criteria through full-text review by the author. Adjudication was performed by the author and was not blinded to the pipeline decision; the source reviews' own inclusion rationale was consulted where available, and unresolved ambiguity was resolved against the prespecified eligibility criteria rather than by modifying criteria post hoc.

A tiered benchmarking strategy quantified screening reliability, extraction integrity, and synthesis validity across the three targets. Screening decisions were benchmarked against the inclusion/exclusion sets of each reference review. I computed recall (proportion of reference-included studies retained by the pipeline), precision (proportion of retained studies that were reference-included), accuracy ((TP + TN)/all retrieved records), and F1 score. Ninety-five percent confidence intervals (95% CIs) for recall, precision, and accuracy were calculated using the Wilson score method.

Extraction and synthesis were assessed using high-level concordance criteria tailored to each domain. For ketamine-neuroimaging, concordance focused on (i) alignment of dominant directionality for network-level motifs, and (ii) agreement with the expert narrative regarding the major qualitative structure of findings (e.g., network-level increases/decreases/normalization patterns). For clozapine-suicidality, concordance emphasized whether the pipeline reproduced the reference review’s core clinical conclusions (protective effect signals during treatment; discontinuation-associated risk) and whether extracted outcomes (ideation/attempt/death/composite) and direction (benefit/harm/neutral) were consistent with the reference narrative where explicitly stated in abstracts. For clozapine perspectives, concordance focused on recovery of the major thematic dimensions reported by the review (benefits, burden/side effects, monitoring barriers, adherence/attitudes, respondent type) and methodological classification (qualitative vs questionnaire-based) when available from abstracts. Across all domains, I also tracked missingness rates for fields known to be abstract-sparse and required that absent information be represented as not reported (NR) rather than imputed, as a safeguard against synthetic completeness.

## Results

Ketamine-neuroimaging: screening performance

Using the same PubMed query and time window as the reference review [15], retrieval yielded 157 records (vs 126 reported in the original paper); 47 were retained after metadata filtering and LLM screening (Tables 1, 2). The larger retrieval set likely reflected database updating and/or indexing changes after the original search date. Using the original published set of 41 included studies as reference, the pipeline retained all original studies and six additional records. Manual adjudication showed that five of these additional records were eligible, and one was ineligible (a protocol). After adjudication, the audit-adjusted reference set therefore comprised 46 eligible studies among 157 records retrieved in the replicated search. The pipeline yielded 46 true positives, one false positive, no false negatives, and 110 true negatives, corresponding to recall 100.0% (46/46; 95% CI: 92.3-100.0%), accuracy 99.4% (156/157; 95% CI: 96.5-99.9%), precision 97.9% (46/47; 95% CI: 88.9-99.6%), and F1 98.9%.

**Table 1:** Network-level concordance between the LLM-derived evidence map and the reference systematic review Detailed concordance between the LLM-derived evidence map and the corresponding sections of the reference systematic review [15]. DMN = Default Mode Network; FPN = Frontoparietal Network; SN = Salience Network; SCN = Subcortical Network; mPFC = medial prefrontal cortex; pgACC = pregenual anterior cingulate cortex; PCC = posterior cingulate cortex; DLPFC = dorsolateral prefrontal cortex; SMA = supplementary motor area; rACC = rostral anterior cingulate cortex; sgACC = subgenual anterior cingulate cortex; dACC = dorsal anterior cingulate cortex; OFC = orbitofrontal cortex; IFG = inferior frontal gyrus; SLF = superior longitudinal fasciculus; ILF = inferior longitudinal fasciculus; UF = uncinate fasciculus; IC = internal capsule; PAG = periaqueductal gray; VTA = ventral tegmental area; NAcc/Nucleus Accumbens = nucleus accumbens; White Matter = cerebral white matter; Task fMRI = task-based functional magnetic resonance imaging; DWI = diffusion-weighted imaging; T1WI = T1-weighted structural imaging; Gold regions/signals/directions = manually defined or reference regions, number of signals, and corresponding directionality; Extracted regions/modalities/signals/directions = automatically or systematically extracted regions, imaging modalities, number of signals, and corresponding directionality; Increased = higher activity, volume, connectivity, diffusivity, or related imaging measure depending on modality; Decreased = lower corresponding imaging measure; Mixed = discordant directions within the same category; Normalized = treatment-related shift toward control-like or clinically normalized values. For display, pre–post change and response-related change were collapsed into ‘Treatment related’, and DWI, T1WI, and task-fMRI findings were collapsed to the modal direction within each network. Concordance ratings reflect thematic network-level directionality and emphasis, not effect sizes or coordinate-level localization.

Role	Network	Reference review (gold standard)				LLM-extracted				
		Regions	Signals (n)	Directions	Direction (collapsed)	Regions	Modalities	Signals (n)	Directions	Direction (collapsed)
Baseline predictor	DMN	mPFC; pgACC	2	Decreased; Increased	Mixed	PCC; pgACC	Task fMRI	2	Decreased; Increased	Mixed
Baseline predictor	FPN	—	0	—	—	DLPFC; SMA	Task fMRI	2	Decreased; Increased	Mixed
Baseline predictor	SN	—	0	—	—	rACC	T1WI	1	Increased	Increased
Baseline predictor	Limbic	Cingulum; Hippocampus; sgACC	3	Increased; Mixed	Increased	Amygdala; Hippocampus; White_Matter	DWI; T1WI	3	Decreased; Increased	Increased
Baseline predictor	SCN	Striatum; Thalamus	2	Decreased; Increased	Mixed	Thalamus	T1WI	1	Increased	Increased
Treatment related	DMN	Forceps; PCC; mPFC; pgACC	4	Decreased; Increased	Increased	mPFC	Task fMRI	1	Normalized	Normalized
Treatment related	FPN	DLPFC; SLF	2	Decreased; Increased; Mixed	Decreased	DLPFC; IFG; Parietal; SLF; SMA	DWI; T1WI; Task fMRI	5	Decreased; Increased; Normalized	Decreased
Treatment related	SN	Insula; dACC	2	Decreased; Increased	Mixed	Insula	Task fMRI	1	Decreased; Increased	Decreased
Treatment related	Limbic	Amygdala; Hippocampus; ILF; Nucleus_Accumbens; OFC; Temporal; UF; sgACC	8	Decreased; Increased; Mixed	Increased	Amygdala; Hippocampus; OFC; sgACC	DWI; T1WI; Task fMRI	4	Decreased; Increased	Increased
Treatment related	SCN	IC; Striatum	2	Increased	Increased	IC; Nucleus_Accumbens; PAG; Striatum; Thalamus; Ventral_Tegmental_Area	DWI; T1WI; Task fMRI	6	Decreased; Increased	Increased

**Table 2:** Pooled performance summary of the LLM pipeline across three validation benchmarks LLM title/abstract screening and selection performance of the pipeline across three reference systematic reviews. Definitions of the screening stage and discrepancy auditing procedures are reported in the text. Metric = evaluation measure used to quantify screening/classification performance; Final retained after screening = number of records kept as relevant after the screening process; audit-adjusted reference n = final reference set size after manual audit/adjudication, including relevant records missed or not present in the original reference standard; TP = true positives, records correctly retained as relevant; FP = false positives, records incorrectly retained as relevant; TN = true negatives, records correctly excluded as irrelevant; FN = false negatives, relevant records incorrectly excluded; Recall = TP/(TP+FN), proportion of all truly relevant records that were retrieved; Precision = TP/(TP+FP), proportion of retained records that were truly relevant; Accuracy = (TP+TN)/(TP+FP+TN+FN), proportion of all classifications that were correct; F1 = harmonic mean of precision and recall, calculated as 2×Precision×Recall/(Precision+Recall). All performance metrics (recall, precision, accuracy, F1) are reported as percentages. Performance is reported against the audit-adjusted reference set (i.e., after manual full-text adjudication of discrepant records); the corresponding originally published reference inclusion sets comprised 41 (ketamine/neuroimaging), 41 (clozapine/suicidality), and 27 (clozapine/perspectives) studies. Wilson score 95% confidence intervals for the audit-adjusted estimates were—ketamine/neuroimaging: recall 92.3–100.0%, precision 88.9–99.6%, accuracy 96.5–99.9%; clozapine/suicidality: recall 67.2–87.7%, precision 61.0–82.4%, accuracy 89.8–94.9%; clozapine/perspectives: recall 78.8–94.5%, precision 68.0–86.8%, accuracy 97.6–98.9%.

Metric	Ketamine / neuroimaging	Clozapine / suicidality	Clozapine / perspectives
Records retained after screening (pipeline) / audit-adjusted reference set size (n)	47/46	63/58	71/63
TP/FP/TN/FN	46/1/110/0	46/17/324/12	56/15/1304/7
Recall/Precision/Accuracy/F1 (%)	100.0/97.9/99.4/98.9	79.3/73.0/92.7/76.0	88.9/78.9/98.4/83.6
Performance summary	Maximal recall with high precision in the audit-adjusted analysis.	Recall limited by abstract availability/reporting.	Performance was boundary-dependent, reflecting the breadth and weak operationalization of the target construct.

Ketamine-neuroimaging: extraction and evidence mapping

**Figure 2:**
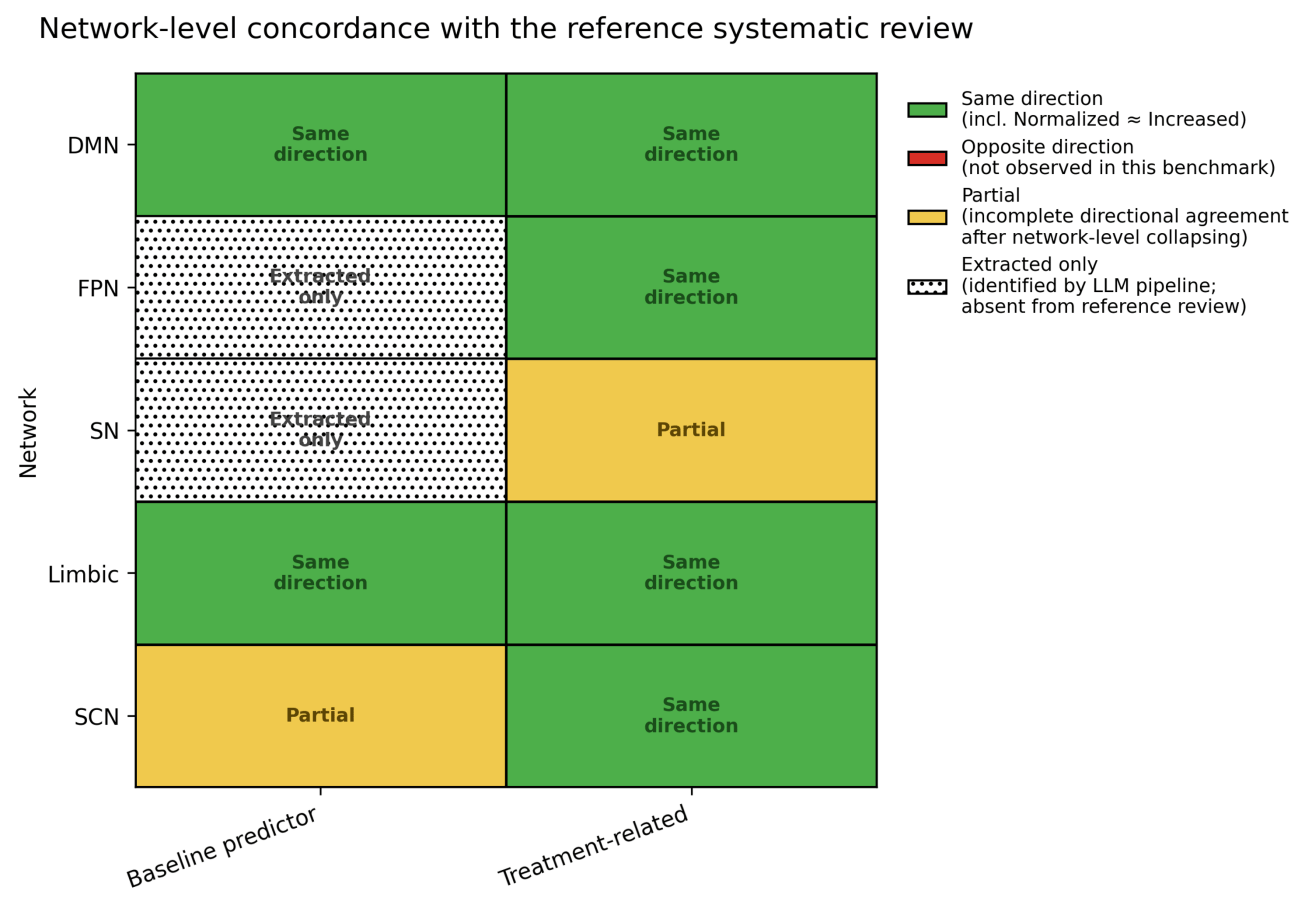
Network-level concordance with the reference systematic review. DMN = Default Mode Network; FPN = Frontoparietal Network; SN = Salience Network; Limbic = Limbic Network; SCN = Subcortical Network. Each cell compares the collapsed reference-review directionality and the collapsed LLM-extracted directionality for one network and one role.

**Figure 3:**
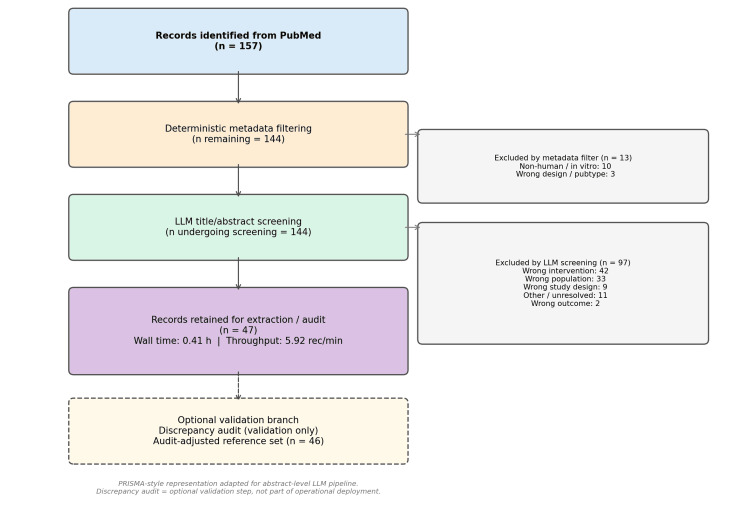
Identification and screening of records - ketamine / neuroimaging

LLM screening of the 144 records to screen was completed in a wall time of 0.41 h (mean throughput: 5.92 records/min; inter-record latency: p50: 10 s, p90: 12 s, p99: 13 s). For included studies, schema-constrained extraction recovered all audited abstract-level descriptors without detected corruption (PMID, sample size, diagnosis, dosing route/frequency). As expected, fields typically confined to full text showed non-trivial missingness and were returned as NR rather than imputed. Abstract-derived synthesis reproduced the major topics of the reference narrative at network/ROI-family level, with divergence largely confined to context-conditional findings (e.g., baseline predictors vs change; task/valence dependence) (Table 1, Figures 2, 3).

Clozapine-suicidality: screening performance and validation

**Figure 4:**
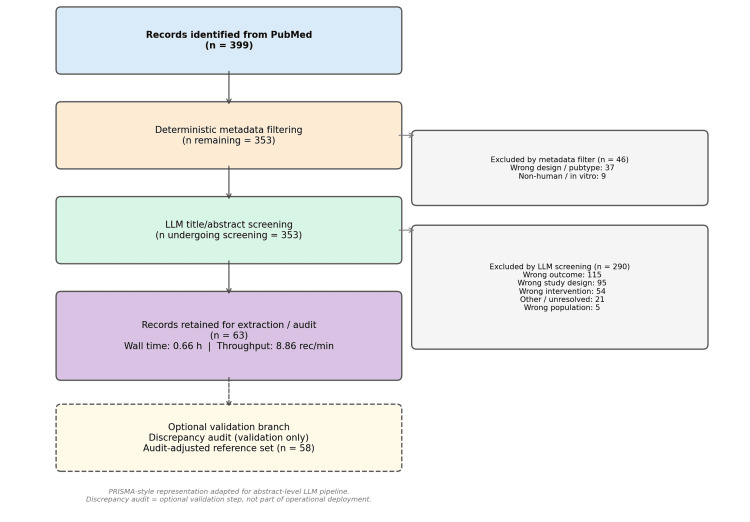
Identification and screening of records - clozapine / suicidality

Using the reference review query constraints [16], 63 records were retained from 399 after the full screening pipeline (wall time: 0.66 h; mean throughput: 8.86 records/min; inter-record latency: p50: 6 s, p90: 11 s, p99: 21 s) (Table 2, Figure 4). Discrepancy audit indicated that discordance was predominantly driven by abstract-level information barriers, including missing abstracts and lack of explicit reporting of suicidality outcomes. Among retained records, 17 did not report direct findings on clozapine and suicidality (e.g., toxicity or non-specific behavioral outcomes). Of the studies included in the original review but not retrieved here, 10 referred to non-suicidal self-injury, which was inconsistently handled between search strategy and eligibility criteria in the reference review, and therefore, they were either not retrieved by the reference string search or excluded in the screening, leading to 12 studies included in the original review but not retrieved here. In addition, 17 studies met the stated eligibility criteria but were not included in the original review, while 29 records overlapped between approaches.

Based on an adjudicated reference set comprising 58 studies (41 original plus 17 additionally eligible studies on suicidal behaviors), the screening pipeline yielded a recall of 79.3% (46/58; 95% CI: 67.2-87.7%), precision of 73.0% (46/63; 95% CI: 61.0-82.4%), accuracy of 92.7% (370/399; 95% CI: 89.8-94.9%), and an F1 score of 76.0%.

Despite incomplete overlap at the record level, the abstract-based synthesis reproduced the main clinical signal of the reference review, namely a protective association during treatment and a signal of increased risk after discontinuation. Within the adjudicated set (n = 58), 38 studies supported a beneficial association, 14 reported no clear association, and a small minority (6) suggested an adverse association; the latter were predominantly attributable to ambiguity or misclassification of outcomes at the abstract level (e.g., toxicity or non-specific behavioral endpoints).

Clozapine patient/caregiver perspectives: screening performance

**Figure 5:**
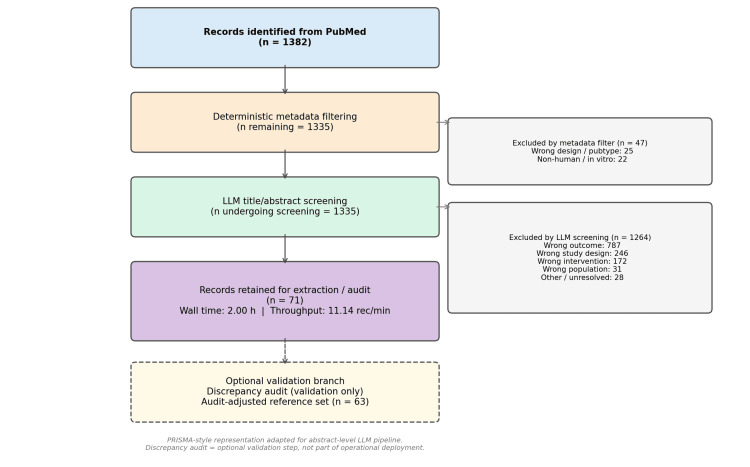
Identification and screening of records - clozapine / patient-caregiver perspectives

Using the reference constraints ([17]; included n = 27), 71 records were retained after LLM title/abstract screening out of 1382 records (Table 2, Figure 5). This benchmark had the longest runtime (wall time: 2.00 h; mean throughput: 11.14 records/min, 668.3 records/hour; inter-record spacing including overhead: p50 5 s, p90 8 s, p99 13 s). Of the 27 studies included in the original review, 20 were correctly identified; the remaining seven were not included, predominantly because of missing abstracts or insufficient information at the abstract level. The target construct in the reference review, patient and/or caregiver or family-member perspectives on clozapine, was broad and only weakly operationalized, encompassing heterogeneous domains such as attitudes, experiences, satisfaction, and treatment-related views. This conceptual ambiguity likely contributed to discordant classification, particularly among false positives: 15 retained records mentioned clozapine but did not clearly assess patient, caregiver, or family perspectives. In addition, full-text adjudication identified 36 further studies meeting the stated eligibility criteria but not included in the original review, yielding an expanded adjudicated reference set of 63 included studies. Against this expanded reference standard, the screening pipeline yielded 56 true positives, 15 false positives, and seven false negatives, corresponding to recall 88.9% (56/63; 95% CI: 78.8-94.5%), precision 78.9% (56/71; 95% CI: 68.0-86.8%), accuracy 98.4% (1360/1382; 95% CI: 97.6-98.9%), and F1 score 83.6%. Despite incomplete record-level overlap, the abstract-derived synthesis reproduced the main narrative clinical conclusions of the reference review. Across extracted records, patient/caregiver perspectives were defined as mostly positive toward clozapine (40/71), with fewer neutral or mixed reports (15/71) and negative reports (16/71).

## Discussion

This study benchmarked a fully local, zero-shot, schema-constrained LLM pipeline for title/abstract screening and abstract-based extraction and synthesis under reproducibility and privacy constraints on consumer-grade hardware. Across three psychiatric benchmarks, performance tracked the density and explicitness of the source text: when the construct of interest was explicitly encoded in abstracts (ketamine-neuroimaging), the pipeline achieved maximal sensitivity with high precision and produced a conservative error profile dominated by over-inclusion; when outcomes were inconsistently reported at abstract level (clozapine-suicidality; clozapine experiences), screening and synthesis were predictably rate-limited by missing or underspecified inputs (Table 2).

The present evaluation should be understood as proof-of-concept workflow benchmarking against three published systematic reviews, rather than as fully independent external validation against a prospective gold standard. The three benchmarks were not a random sample of psychiatry domains, single-author adjudication was used without blinded dual review, and the audit-adjusted reference sets were partly modified by the same investigator who developed the pipeline. These constraints limit the strength of inferences that can be drawn from the reported metrics. Many prior LLM-assisted review implementations emphasize isolated stages (retrieval and/or screening) or depend on proprietary cloud models, limiting deployability under strict governance and reproducibility requirements [1,3]. In contrast, the present work operationalizes a local-first workflow that couples screening with structured abstract-level extraction and high-level synthesis using fixed prompts and schemas. Because this study was not designed to compare models, no inference about model superiority should be drawn from these results.

In the ketamine benchmark, discrepancy auditing showed that strict false positives were largely predictable confounds (e.g., protocol records), whereas other discordant items were plausibly eligible but absent from the reference set. This profile is favorable for evidence surveillance: in living or rapid scoping use cases, a small manual triage burden is generally preferable to missed eligible studies. Beyond selection, abstract-derived extraction and ROI/network aggregation reproduced the backbone of the reference narrative, with divergence concentrated in context-conditional signals that abstracts often compress (e.g., baseline predictors vs change; task/valence dependence). This boundary is intrinsic to abstract-only synthesis: it supports field-level “heat mapping” but not coordinate-level mechanistic inference.

In the clozapine benchmarks, most discordance reflected abstract-level information barriers (missing abstracts; outcomes not stated), rather than fabrication. In these domains, full-text access would be expected to improve sensitivity, but at the cost of additional operational complexity and potential parsing/section-level misclassification risks [18]. A pragmatic deployment model is therefore hybrid: high-throughput abstract-level mapping for coverage, paired with periodic human audits and targeted full-text deep dives where abstract informativeness is low or clinical stakes are high [1,19].

A consistent methodological trade-off emerged across the three targets: abstract-only processing entails partial information loss relative to full text [20-22], but yields major gains in the feasibility of retrieval, governance, and scalability well beyond human limits. Abstracts routinely omit method-critical details (e.g., preprocessing, analytic thresholds, full timepoint structures), compress conditional findings, and may omit clinically decisive outcomes entirely; however, omissions are often related to methods rather than results [22]. Moreover, manual screening itself is associated with non-negligible error rates [23].

Several limitations remain. Reliance on abstracts alone precludes the extraction of granular methodological details and does not permit a formal risk-of-bias assessment. False positives could inflate or distort the synthesized signal; nonetheless, in the present analysis, the main thematic and directional conclusions were robust to their inclusion. In addition, the absence of manual reference-list screening of included studies may reduce sensitivity in areas where indexing is incomplete or abstracts are uninformative; this step can be added as an optional augmentation when higher recall is required. For imaging outcomes, the ROI-to-network mapping layer applies a simplifying ontology that facilitates synthesis but may obscure edge-level specificity. More broadly, parameter choices, prompt design, and schema constraints encode domain assumptions; while detailed prompting is increasingly standard in LLM-based evidence synthesis, these choices can introduce subjectivity if not explicitly documented and stress-tested [24]. Residual hallucination risk also remains [25,26], although schema enforcement (constrained enumerations rather than free generation) and conservative missingness handling (returning ‘NR’) were designed to minimize fabrication and performed well in audited fields. Because the model may have been pretrained on biomedical abstracts and possibly on the benchmark reviews themselves, contamination cannot be excluded. However, the observed discrepancy structure argues against simple memorization of published inclusion tables: the pipeline both missed some reference-included records and retained records adjudicated as eligible but absent from the reference reviews. The benchmark should therefore be interpreted as workflow-level external replication under potential corpus familiarity, not as a contamination-free prospective evaluation. A further consideration is that the reference standard was partly audit-adjusted by the same investigator who developed and tested the pipeline; while the adjudication procedure was guided by pre-specified eligibility criteria, single-author adjudication without blinding may introduce incorporation bias or reviewer expectancy effects, and this limitation is most consequential in benchmarks where the audit-adjusted reference set differs substantially from the originally published inclusions. This study was not designed as a model-comparison or ablation study; therefore, the reported performance should be interpreted as proof-of-concept benchmarking of one reproducible local workflow under fixed hardware constraints, not as evidence that the selected model, prompt, or schema is optimal. The unit of benchmarking was the review-level workflow rather than individual abstract classification alone; the number of benchmark reviews was small, and the findings should be interpreted as proof-of-concept external benchmarking across three psychiatry use-cases rather than broad validation. Generalizability is therefore limited by the evaluation scope (three psychiatric topics), and generalizability beyond psychiatry has not been established, as benchmarks were selected within the author’s area of methodological and clinical expertise. In addition, I did not quantify human decision variability through independent dual screening/extraction, which precludes estimation of inter-rater reliability for the benchmark. Single-author adjudication was an intentional feature of this proof-of-concept design rather than an oversight; however, the pipeline is fully reproducible, and its results can be independently verified, because it uses an open-weight model with fixed prompts, deterministic decoding, publicly available code, and an append-only audit trail of every screening and adjudication decision. The screening inputs, the adjudication criteria, and the resulting reference set are therefore fully transparent and can be independently re-derived and re-adjudicated by other investigators. This decision-level traceability partially mitigates the absence of dual adjudication, since it exposes every classification to external verification - in contrast to conventional manual dual screening, whose individual decisions are rarely reproducible - although independent dual adjudication remains the appropriate next step to estimate inter-rater reliability. The study also does not include comparisons with traditional keyword/rule-based screening, non-LLM classifiers, alternative open-weight models, or human-assisted screening baselines under the same retrieval conditions; therefore, the results demonstrate that the pipeline can function, but do not establish whether it performs better than simpler or cheaper alternatives. Finally, local deployment avoids transmission of records to cloud APIs and permits direct measurement and control of inference hardware; however, this study did not quantify net energy consumption or carbon footprint, which should be measured directly in future deployment studies.

## Conclusions

In conclusion, this study provides proof-of-concept benchmarking of a fully local, zero-shot, schema-constrained pipeline for systematic scoping reviews, extending prior work that has largely focused on LLM-assisted screening. Across three psychiatric use-cases, retrieval and synthesis were conservative and directionally consistent, supporting the use of local LLMs for scalable evidence surveillance and high-level evidence mapping where target signals are explicitly reported in abstracts. The central trade-off is reduced mechanistic resolution relative to full-text synthesis; a pragmatic deployment model is therefore hybrid, coupling high-throughput abstract-level mapping with periodic human audits and targeted full-text review where uncertainty or clinical impact is highest.

## Appendices

**Table 3:** Detailed prompts for the review: Neural correlates of treatment response to ketamine for treatment-resistant depression: A systematic review of MRI-based studies

Search Query:
("ketamine"[MeSH Terms] OR "ketamine"[All Fields] OR "esketamine"[All Fields]) AND ("depressive disorder"[MeSH Terms] OR "depression"[All Fields] OR "depressive"[All Fields]) AND "magnetic resonance"[All Fields] AND 2018/01/01:2024/05/31[Date - Publication]
Selection criteria:
OBJECTIVE
Identify primary human studies investigating brain MRI (structural, functional, or perfusion) as a correlate or predictor of clinical response to ketamine/esketamine treatment in depression.
1. POPULATION (Strict Inclusion)
Inclusion: Human participants with a primary diagnosis of a Depressive Disorder (MDD, TRD, Bipolar Depression, or Major Depressive Episode).
Note on Comorbidity: Include studies where depression is the primary focus, even if comorbid symptoms (e.g., anxiety, suicidal ideation) are present.
Note on Controls: Presence of Healthy Controls (HC) is irrelevant for exclusion; only the absence of a depressed clinical group is grounds for exclusion.
Exclusion: Studies focusing exclusively on other disorders (e.g., Schizophrenia-only, PTSD-only, Alcohol Use Disorder-only) without a depressive disorder subgroup.
2. INTERVENTION (Therapeutic Intent)
Inclusion: Therapeutic administration of Ketamine (racemic, R, or S/esketamine) aimed at symptom reduction. Any route (IV, IN, Oral, IM).
Exclusion: "Ketamine Challenge" studies in healthy volunteers to model psychosis. Ketamine used solely as an anesthetic in non-psychiatric surgery. Recreational use/Abuse cohorts (unless specific antidepressant response is measured). Binding/Radiochemistry-only studies (e.g., PET-ligand development without clinical MRI outcome).
3. MRI MODALITY (Technical Precision)
Inclusion: At least one MRI-based measure. Structural: T1-weighted (VBM, Cortical Thickness, Volumetry, Shape analysis). Diffusion: DWI/DTI/DKI/NODDI (FA, MD, Neurite density). Functional: T2-weighted fMRI (Resting-state, Seed-based FC, ICA, Graph theory, Task-based activation). Perfusion: ASL (Arterial Spin Labeling) or DSC.
The "Multimodal" Rule (CRITICAL): Do NOT exclude if MRI is combined with other modalities (EEG, PET, MRS, MEG, Blood biomarkers). As long as MRI data is reported, the study is ELIGIBLE.
Exclusion: Studies using only non-MRI methods (e.g., 1H-MRS only, EEG-only, PET-only).
4. OUTCOME & ANALYTIC TARGET (Scope)
Inclusion: Study must link MRI data to ketamine-induced clinical change or baseline prediction.
(A) Longitudinal: Comparison of MRI metrics Pre- vs. Post-ketamine.
(B) Correlational: Brain change Delta correlated with symptom change Delta.
(C) Predictive: Baseline (Pre-treatment) MRI features predicting subsequent response/remission.
Exclusion: MRI used only for screening/safety without reporting brain-response associations.
5. STUDY DESIGN & PUBLICATION TYPE
Inclusion: Randomized Controlled Trials (RCTs), Open-label trials, Longitudinal observational studies, Case series (N > 1).
Exclusion: Case reports (N=1), Systematic/Narrative Reviews, Meta-analyses (as they are not primary data), Editorials, Conference abstracts (unless sufficient data is provided), and Animal/Preclinical studies.
LLM OPERATIONAL INSTRUCTIONS (Decision Logic)
Zero-Inference Rule (Animal vs. Human): If the abstract mentions "patients," "participants," "subjects," or "recruited," and cites a clinical diagnosis (e.g., MDD), assume Human. Do not exclude based on the word "animal" if it appears in the discussion or background (e.g., "consistent with animal models").
The "MRS/Spectroscopy" Trap: LLMs often see "1H-MRS" and exclude. You must verify if fMRI or T1-weighted imaging is also mentioned. If MRI + MRS are both present INCLUDE.
Presumption of Inclusion (Abstract Stage): If the abstract states "MRI was performed" and "ketamine was administered to patients," but doesn't explicitly report the correlation coefficient or p-value in the text INCLUDE.
Bipolar Inclusion: Given the high frequency of TRD studies including Bipolar II or Bipolar Depression, do not exclude unless the study is purely about Mania or Bipolar I in an euthymic state.
FINAL DECISION MATRIX
INCLUDE: Meets all 5 criteria.
EXCLUDE: Explicitly fails at least one criterion (e.g., N=1, No MRI, No Ketamine).

**Table 4:** Detailed prompts for the review: Prevention of suicide by clozapine in mental disorders: systematic review

Search Query:
("clozapine"[MeSH Terms] OR "clozapine"[All Fields]) AND (suicid*[All Fields] OR self-injury[All Fields]) AND 1970/01/01:2022/07/17[Date - Publication]
Selection criteria:
OBJECTIVE
Identify human clinical evidence on the association between clozapine administration and suicidality outcomes (ideation, attempts, completed suicide), across clinical conditions. Retrospective evidence is included. Additionally, include systematic-search review papers as secondary evidence sources.
1. POPULATION (Strict Inclusion)
Inclusion: Human participants receiving clozapine for any psychiatric condition.
Controls are not required. Comparator arms (other antipsychotics, treatment-as-usual) are optional.
Exclusion:
Non-human / preclinical studies.
Studies where clozapine is not actually administered to the patients being analyzed.
2. INTERVENTION / EXPOSURE (Clozapine Administration)
Inclusion: Any clozapine administration intended for clinical treatment (initiation, continuation, switch to clozapine, augmentation) in any setting, any dose, any duration.
Exclusion: Papers that merely mention clozapine but do not analyze a clozapine-exposed patients (e.g., background/pharmacology only).
Pure pharmacokinetic/pharmacodynamic studies with no suicidality outcome data.
Case reports with no analyzable suicidality information (see design rules below).
3. OUTCOME (Suicidality, Baseline-to-Post Change Required)
Inclusion (CRITICAL): Must report suicidality outcomes for the clozapine-treated patients, covering at least one of: Suicide ideation, Suicide attempts, Completed suicide
Exclusion: Papers reporting only general “mortality” without suicide-specific outcomes, unless suicide is explicitly separated.
4. STUDY DESIGN & PUBLICATION TYPE
Primary research inclusion: RCTs, pragmatic trials, open-label trials, prospective/retrospective studies, case-control studies, registry/database studies, mirror-image studies, case series and case reports.
Primary research exclusion: Conference abstracts, letters without primary data sufficient to extract baseline/post suicidality. Protocols.
Secondary evidence inclusion (reviews): Review papers are included only if they report a systematic search of database(s).
Secondary evidence exclusion: Narrative reviews, mini-reviews, opinion pieces.
5. LLM OPERATIONAL INSTRUCTIONS (Decision Logic)
Zero-Inference Rule (Human vs non-human): If the abstract describes patients/participants and a diagnosis, treat as human unless explicitly animal/preclinical.
Systematic Review Test: If it’s a review, INCLUDE only when there is an explicit database search (named databases, search terms/timeframe, PRISMA-ish flow, or at minimum a described systematic search process). Otherwise EXCLUDE as narrative/mini.
FINAL DECISION MATRIX
INCLUDE: Human clozapine-treated + diagnosis recorded + treatment suicidality outcome(s) reported + eligible design/type.
EXCLUDE: Explicitly fails any of the above (no clozapine exposure group, no diagnosis info, non-human, narrative/mini review).

**Table 5:** Detailed prompts for the review: Patient and caregivers perspective about clozapine: A systematic review

Search Query:
("clozapine"[MeSH Terms] OR clozapine[All Fields]) AND (patient[All Fields] OR participant*[All Fields] OR caregiver[All Fields] OR family[All Fields] OR consumer[All Fields]) AND (knowledge[All Fields] OR attitude[All Fields] OR adherence[All Fields] OR perception[All Fields] OR satisfaction[All Fields] OR discontinuation[All Fields]) AND 1970/01/01:2023/03/31[Date - Publication]
Selection criteria:
OBJECTIVE
Identify human clinical evidence (original research and review articles) exploring the lived experience of using clozapine, from the perspective of patients and/or caregivers/family members, irrespective of psychiatric diagnosis.
1. POPULATION (Inclusion)
Inclusion: Human participants who are patients using clozapine and/or their caregivers/family members.
Any psychiatric diagnosis (including mixed or unspecified diagnoses).
Exclusion: Non-human / preclinical studies.
2. PHENOMENON OF INTEREST / EXPOSURE (Clozapine Use)
Inclusion: Any experience related to using clozapine (e.g., initiation, continuation, switching, adherence, monitoring, side effects, perceived benefits, burdens, decision-making, service delivery).
Any dose, duration, setting, and care model.
Exclusion: Papers that only mention clozapine as background/pharmacology with no patient/caregiver/family experience data.
Pure pharmacokinetic/pharmacodynamic studies with no experiential component.
3. OUTCOME (Experience Data)
Inclusion (CRITICAL): The article must report or analyze or synthesize patient and/or caregiver/family member experiences with clozapine. Examples include:
Qualitative findings (themes, narratives, interviews, focus groups, ethnography)
Quantitative experience-related data (surveys, patient-reported experience measures, satisfaction, attitudes, preferences, burden/quality-of-life explicitly tied to clozapine use)
Mixed-methods experience outcomes
Exclusion: Clinical efficacy/safety-only studies that do not report any patient/caregiver experience.
4. STUDY DESIGN & PUBLICATION TYPE
Inclusion: Original research (qualitative, quantitative, mixed-methods).
Review articles (qualitative or quantitative) that explore/summarize patient and/or caregiver/family experience with clozapine.
Exclusion: Case reports / case series.
Conference abstracts/papers.
Editorials, commentaries, letters without primary experience data.
5. LLM OPERATIONAL INSTRUCTIONS (Decision Logic)
Zero-Inference Rule (Human vs non-human): If the abstract describes patients/participants/caregivers/family members, treat as human unless explicitly animal/preclinical.
Experience Test (CRITICAL): INCLUDE only if the abstract indicates experiential content (e.g., interview/survey/attitudes/preferences/satisfaction/burden) or a review explicitly focused on experience with clozapine.
Publication Type Filter: EXCLUDE if it is clearly a case report/case series, conference item, or editorial/commentary/letter without primary experience data.
FINAL DECISION MATRIX
INCLUDE: Human patients and/or caregivers/family members + clozapine use + experiential data (or review focused on experience) + eligible publication type.
EXCLUDE: Non-human; no experiential component; case report/case series; conference paper/abstract; editorial/commentary/letter without primary experience data.
